# A novel variation of *SERPINC1* caused deep venous thrombosis in a Chinese family: A case report

**DOI:** 10.1097/MD.0000000000013999

**Published:** 2019-01-04

**Authors:** Yu Peng, Tun Wang, Yu Zheng, Aojie Lian, Di Zhang, Zhimin Xiong, Zhengmao Hu, Kun Xia, Chang Shu

**Affiliations:** a Pediatrics Research Institute of Hunan Province, Hunan Children's Hospital, Changsha; b Center for Medical Genetics & Hunan Key Laboratory of Medical Genetics, School of Life Sciences, Central South University; c Department of Vascular Surgery, The Second Xiangya Hospital of Central South University; d Clinical Laboratory, The Third Xiangya Hospital of Central South University, Changsha, Hunan, China.

**Keywords:** antithrombin, deep venous thrombosis, missense mutation, *SERPINC1*

## Abstract

**Rationale:**

Deep vein thrombosis (DVT) is the formation of a blood clot formed in the deep veins of the lower limbs. Known genetic factors of DVT include deficiencies of antithrombin (AT), protein C, protein S, factor V Leiden mutation, and prothrombin G20210A mutation. Here, a 5-generation Chinese family with inherited DVT was recruited for genetic analysis.

**Patient concerns:**

The patient came to see a doctor because of leg swelling. A color Doppler ultrasound examination showed extensive thrombosis within the deep veins of her left leg. Computed tomography angiography showed a pulmonary embolism in her right lower pulmonary artery.

**Diagnoses:**

Type II AT deficiency lead to inherited DVT.

**Interventions:**

Whole-exome sequencing and cosegregation analysis were carried for the DVT family.

**Outcomes:**

An unreported heterozygous missense variation, c.281T>C, was identified within the *SERPINC1* gene. This missense variation of *SERPINC1* leads to type II AT deficiency.

**Lessons:**

This result further enriched the variation spectrum of the *SERPINC1* gene.

## 1 Introduction

Deep vein thrombosis (DVT) is the formation of a blood clot formed in the deep veins of the lower limbs. A venous thrombus can become a life-threatening pulmonary embolism (PE), if a clot formed in the deep veins breaks free and enters the arteries of the lungs.^[1]^ PE is a primary cause of mortality associated with DVT.^[2]^ Known genetic factors of DVT include deficiencies of antithrombin (AT), protein C, protein S, factor V Leiden mutation, and prothrombin G20210A mutation.^[2,3]^ AT deficiency is a major risk factor for venous thromboembolic disorders. Inherited AT deficiency was 1st described by Egeberg in a Scandinavian venous thromboembolism family in 1965. It is rare, with a prevalence between 0.2% and 0.02% in the general population.^[4,5]^ Some AT deficiency patients also show mesenteric venous thrombosis.^[6,7]^ Blood clots formed in other uncommon sites, such as the cerebral sinus and the portal, hepatic, renal, and retinal blood vessels have also been reported.^[8–10]^

The AT, a major inhibitor of blood coagulation in plasma, is a potent inactivator of factor Xa (FXa), thrombin, and other cofactors. AT concentration in the reference plasma is about 150 μg/mL.^[8,11,12]^ The concentration in normal population ranging from 150 to 400 μg/mL.^[13]^ AT protein belongs to the serine protease inhibitor (SERPIN) family and is synthesized predominantly in hepatocytes. The human AT gene (*SERPINC1*, OMIM: 107300) encodes the AT precursor, which has 464 amino acids, of which 32 amino acids form a signal peptide at the N-terminus. Mature AT protein has 2 important functional domains, which include the heparin binding-site domain located at the N-terminus and the reactive site domain located at the C-terminus. The anticoagulant effect of AT can be accelerated at least a thousand-fold in the presence of the unique sequence-specific pentasaccharide domain of heparin or heparan sulfate.^[8]^ Under normal physiologic circumstances, AT activation is usually induced by heparan sulfate proteoglycans, which are located on the inner wall of vascular system. The interaction of AT and heparan sulfate in vivo substantially localizes the function of AT to inhibition of the serine proteases of the coagulation cascade within the bloodstream, allowing their coagulant activity in damaged tissue outside the vascular system.^[14]^ AT deficiency can be classified into type I (quantitative) and type II (qualitative); type I deficiency indicates both AT activity and antigen levels are reduced proportionately, and type II deficiency indicates normal antigen levels associated with low AT activity.

Inherited AT deficiency usually follows an autosomal dominant pattern, although a few recessive cases have occasionally been reported.^[15]^ In this study, we recruited a DVT family with autosomal dominant inheritance. Whole-exome sequencing identified a novel missense variation in *SERPINC1* and segregating with the phenotype in this family.

## 2 Materials and methods

### 2.1 Ethical approval and consent for publication of the data

Ethical approval was not necessary because only peripheral blood was collected from recruited subject for DNA extracted, and written informed consent was obtained before blood collection.

### 2.2 Whole-exome sequencing and data analysis

**Figure 1:**
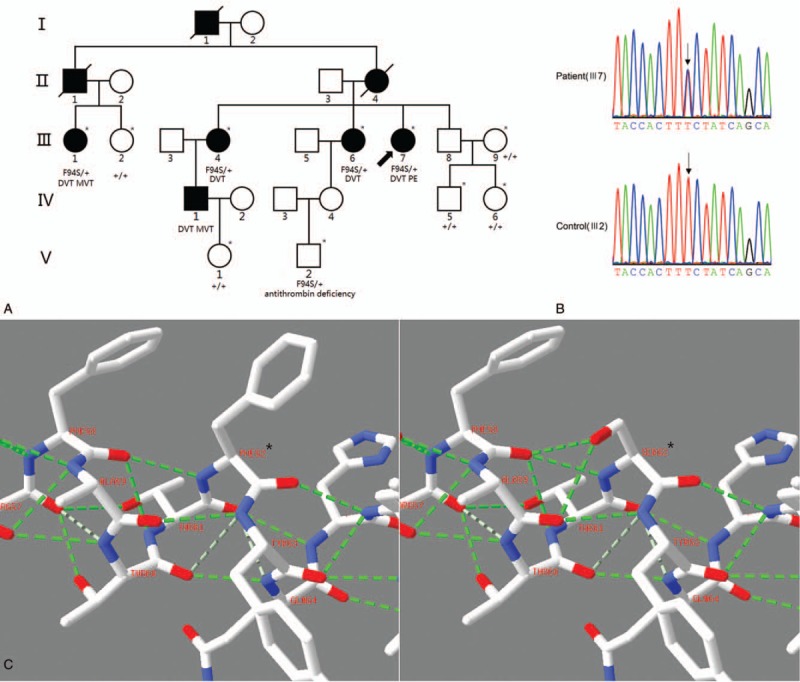
(A) The pedigree of a Chinese deep vein thrombosis (DVT) family (∗ represents the individual's DNA was available). (B) c.281T>C variation in *SERPINC1* gene. (C) The 2 excrescent strong H-bonds formed by Ser62 side chain and main chain amide of Phe58 and Ala59 (red represents O, blue represents N, fluorescent green represents strong H-bond, grayish green represents weak H-bond).

Genomic DNA was extracted from the leukocytes of all recruited individuals (Fig. 1A) in the DVT family by the standard proteinase K digestion and phenol-chloroform method. For the sample chosen for exome sequencing, about 1 μg of genomic DNA was used to construct exome library using the SureSelect Human All Exon V5 Kit (Agilent, Santa Clara, CA) following the manufacturer's instructions. Illumina Hiseq2000 sequencer was employed to sequence the enriched DNA fragments (Illumina, San Diego, CA). FastQC (http://www.bioinformatics.babraham.ac.uk/projects/fastqc/) tool was used to evaluate reads quality and our in-house script was used to filter low-quality reads. The quality passed reads were subsequently mapped to human reference sequence (GRCh37/hg19) by alignment tool Burrows-Wheeler Aligner (BWA, v0.7.10).^[16]^ Genome analysis toolkit (GATK, v3.2)^[17]^ was then employed to call variants.

### 2.3 Sanger sequencing

Sanger sequencing was used to validate the cosegregating status of the mutations through the filtering procedures. Primers were designed by Primer3 program (http://frodo.wi.mit.edu/).

### 2.4 AT activity and antigen level assays

The AT activity was detected using Stago's STA-R Evolution Coagulation Analyzer (Diagnostica Stago, Theale, England) by chromogenic substrate method. AT antigen level was detected using Dirui CS-400B biochemical Analyzer (Changchun, China) by immunoturbidimetric methods. The data was showed in Table 1.

**Table 1:**
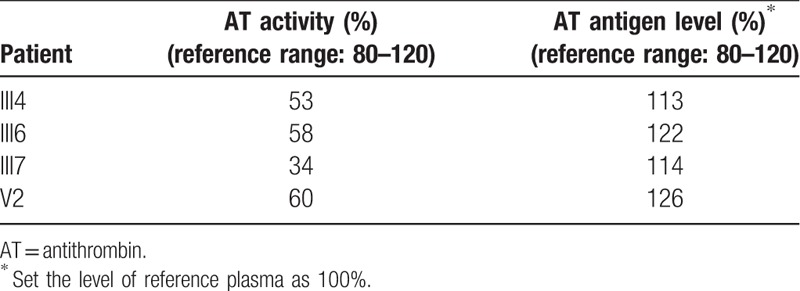
AT activity and antigen level of relevant members in the deep vein thrombosis family.

### 2.5 Bioinformatics prediction and protein structural analysis

Functional prediction and conserved analysis were performed using ANNOVAR^[18]^ (http://annovar.openbioinformatics.org/) (Table 2). The sequence of the mutant AT protein was sent to SWISS-MODEL (http://swissmodel.expasy.org/)^[19]^ for homology modeling. Swiss-PdbViewer (SPDBV)^[19]^ software was used to analyze the structural changes of the mutant AT protein and generate structural figures.

**Table 2:**
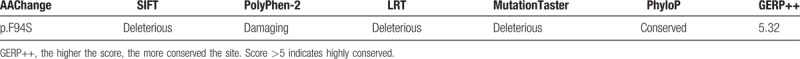
Functional prediction and conservation analysis of *SERPINC1* F94S mutation by ANNOVAR.

## 3 Case report

A Chinese DVT family with autosomal dominant inheritance was recruited in this study (Fig. 1A). All recruited patients underwent detailed clinical examinations including color Doppler ultrasound, computed tomography angiography (CTA), coagulation function at the Second Xiangya Hospital, Central South University. All recruited members underwent blood collection after providing informed consent. We also recruited 205 unrelated controls from Hunan, China. All of the controls had no DVT phenotypes and no family history of venous thrombosis.

### 3.1 Results of the genetic analysis

Totally, 10,981 nonsynonymous SNVs and 346 Indels were identified by WES in the proband (III7) genome. The coding regions of *SERPINC1*, *PROS1*, *PROC*, *F5*, and *F2* were checked and only 1 heterozygous missense variation, c.281T>C, was identified within the *SERPINC1* gene (Fig. 1B). Sanger sequencing showed that the variation was cosegregated with DVT phenotype in the family except for V2 with low AT activity and normal AT antigen level (Table 1). V2 is a 10–year-old boy, consider that the minimum onset age of DVT is 24 years in this family, we suspected that V2 had not reached the onset age of DVT as yet. The variation (c.281T>C) was absent in the genomeAD database (http://gnomad.broadinstitute.org) and is also absent in 205 normal controls. The mutation caused a Phe94Ser (p.F94S) substitution. Because the signal peptide (32 aa) at the N-terminus is cleaved before the AT protein is secreted into the plasma, the variation site was F62S in the mature AT protein. Functional prediction indicated that the F62S variation is damaging or deleterious and the Phe62 was highly conserved during evolution (Table 2). It is indicated that the Phe62 is important for the function of AT. Homology modeling and structural analysis of the F62S variation suggest that it would minimally influence the native conformation of AT protein. Phe is an aromatic group hydrophobic nonpolar amino acid; its side chain forms no H-bonds with other amino acid residues. Ser is a hydroxyl group polar amino acid; its OH-side chain may form 2 additional H-bonds with Phe58 and Ala59 (Fig. 1C).

### 3.2 Diagnosis

The proband (III7) was diagnosed as DVT at the age of 29, the latest recurrence of the thrombotic event at the age of 46. A color Doppler ultrasound examination showed extensive thrombosis within the deep veins of her left leg. No abnormality was found in the abdomen. CTA showed a PE in her right lower pulmonary artery. The coagulation function showed increased fibrin degradation products (20.8 μg/mL, normal: 0–5), D-dimer (6.5 μg/mL, normal: 0–0.55). AT activity and antigen level assays showed the AT activity was 34%, AT antigen level was 171 mg/L. All the patients (III1, III4, III6, and IV1) exhibited DVT in the left lower limb. Subjects III1 and IV1 also showed mesenteric venous thrombosis. Subject IV1 had a history of intestinal necrosis. The onset ages of the 5 patients were 29, 42, 50, 45, and 24 years. AT activity and antigen level data of the other family members are showed in Table 1. Considering the ratio of AT activity to antigen level, we considered it is a type II AT deficiency.

## 4 Discussion

The AT is a key factor of the anticoagulation effect induced by a unique sequence-specific pentasaccharide domain of heparan sulfate in the blood circulation system. The main physiologic function of AT is inhibition of FXa, thrombin, and to a lesser extent, FIXa, FXIa, FXIIa, tissue plasminogen activator (tPA), urokinase, trypsin, plasmin, and kallikrein.^[8,20]^ When AT binds to heparin, the conformational changes of the protein cause a thousand-fold increase in its inhibitory activity. In addition, AT transforms from a low-affinity heparin binding conformation to a high-affinity heparin binding conformation. The heparin binding occurs at the upper half of the D-helix, the base of the A-helix and the amino terminus of the AT protein. The pentasaccharide binds by hydrogen bonding of its sulfates and carboxylates to Arg129 and Lys125 in helix D, Arg46 and Arg47 in helix A, and Lys114, Glu113, Lys11, and Arg13 at the N-terminus.^[14]^

In this study, we identified a heterozygous F62S variation of the *SERPINC1* gene that cosegregated with type II AT deficiency in a Chinese DVT family. Functional prediction analysis revealed that this variation is damaging or deleterious. Conserved analysis and multiple alignment analysis showed that Phe62 is highly conserved in ATs throughout evolution (Table 2). These results indicate that Phe62 is important for AT function.

Phe62 located in the middle of helix A, which contains the residues Arg47 to Ser69 in the key region with which the heparin pentasaccharide primarily interacts. In this region, a missense variation, such as R47C, may cause a heparin binding abnormality resulting in a loss of heparin binding ability.^[15]^ However, other variations in helix A may impair the conformational changes needed for AT activation, such as A54V, S56P,^[21]^ and A59V.^[22]^ A59V even shows normal heparin binding affinity.^[22]^ The process of AT activation has been well studied. It requires several gross conformational changes for initiation, such as helix A rearrangement to expose Arg46 and Arg47, tilting of the helix D to form the 2-turn P-helix and the extension of the C-terminus of helix D.^[23]^ These conformational changes promote the expulsion of the hinge region of the reactive center loop from central β-sheet A, which finally exposes the reactive center loop to the protease.^[23]^ In our case, even if Ser62 did not change the main structure of the AT protein, the 2 excrescent H-bonds formed by the Ser62 OH-side chain and the main chain amide of Phe58 and Ala59 might influence the rearrangement of helix A, impairing the normal allosteric mechanism of the AT protein (Fig. 1C).

The concentration in normal population ranging from 150 to 400 μg/mL.^[13]^ This is quite a wide range, so it is difficult to define whether the AT antigen level of the patients are normal or reduced. In our study, F62S variation may also cause the earlier degradation of the *SERPINC1* mRNA; therefore, both AT activity and antigen level may be reduced, only the proportion is different. This is a limitation of our study.

In conclusion, we have identified a novel missense variation, c.281T>C, within the *SERPINC1* gene in a DTV family. Our findings provide new knowledge of the variation spectrum of AT deficiencies and are important for understanding the heterogeneity of DVT in the Chinese population.

## Acknowledgment

The authors greatly thank all the patients and their family member who participated in this study.

## Author contributions

**Data curation:** Yu Zheng.

**Funding acquisition:** Zhengmao Hu.

**Investigation:** Tun Wang.

**Methodology:** Yu Peng, Zhengmao Hu.

**Project administration:** Kun Xia, Chang Shu.

**Resources:** Tun Wang, Chang Shu.

**Software:** Yu Zheng.

**Validation:** Aojie Lian, Di Zhang, Zhimin Xiong.

**Writing – original draft:** Yu Peng.

**Writing – review & editing:** Yu Peng.
